# Externalizing Behaviors and Alzheimer’s Disease and Any Dementia: A Multigeneration Cohort Study in Sweden

**DOI:** 10.1093/geroni/igad117

**Published:** 2023-10-09

**Authors:** Carmen Solares, Le Zhang, Zheng Chang, Henrik Andershed, Jonas Persson, Henrik Larsson

**Affiliations:** School of Behavioural, Social and Legal Sciences, Örebro University, Örebro, Sweden; Department of Medical Epidemiology and Biostatistics, Karolinska Institutet, Stockholm, Sweden; Department of Medical Epidemiology and Biostatistics, Karolinska Institutet, Stockholm, Sweden; School of Behavioural, Social and Legal Sciences, Örebro University, Örebro, Sweden; School of Behavioural, Social and Legal Sciences, Örebro University, Örebro, Sweden; Aging Research Center, Department of Neurobiology, Care Sciences and Society, Karolinska Institutet, Stockholm, Sweden; Department of Medical Epidemiology and Biostatistics, Karolinska Institutet, Stockholm, Sweden; School of Medical Sciences, Örebro University, Örebro, Sweden

**Keywords:** Criminal behavior, Epidemiology, Family design, Major neurodegenerative disorders, Substance abuse

## Abstract

**Background and Objectives:**

We examined the extent to which externalizing behaviors such as violent and nonviolent criminal behavior, and substance use disorders (SUD) are associate with the onset of Alzheimer’s disease (AD) and any dementia in prior generations.

**Research Design and Methods:**

A nationwide cohort of 2,463,033 individuals born between 1973 and 1997 (index persons) were linked to their biological relatives (parents, grandparents, and uncles/aunts) using Swedish national registers. Cox regression models were used to examine the association between each measure of externalizing behaviors with AD and any dementia in each of the relative cohorts.

**Results:**

Parents of index persons with externalizing behaviors had an increased risk for AD compared with parents of index persons without externalizing behaviors—nonviolent criminal behavior: Hazard Ratio (HR) = 1.16, 95% Confidence Intervals (CI) 1.10–1.22; violent criminal behavior: HR = 1.32 (95% CI: 1.19–1.45); SUD: HR = 1.28 (95% CI: 1.17, 1.40). The associations attenuated with decreasing familial relatedness. Relatives of individuals with externalizing behaviors compared with relatives of individuals without, showed an increased risk of having both early-onset and late-onset AD but the strength of the associations was higher for early-onset AD than for late-onset AD. A similar pattern of results was observed for the association with any dementia.

**Discussion and Implications:**

Externalizing behaviors are associated with AD and any dementia in prior generations. The associations were stronger for parents in comparison with grandparents and uncles/aunts, suggesting shared familial risks between conditions. This warrants further studies examining common genetic and family-wide environmental factors that may contribute to identifying common underlying mechanisms to the development of externalizing behaviors, AD, and any dementia.

**Translational Significance:** In this longitudinal multigeneration study, we explored the familial co-aggregation of externalizing behaviors (i.e., criminal behavior and substance abuse) with Alzheimer’s disease (AD) and any dementia, using data from several Swedish national registers. Results showed that externalizing behaviors are associated with AD and any dementia in prior generations. Associations attenuated with decreasing relatedness (parents, grandparents, and uncles/aunts), suggesting shared familial risk between conditions. The study has important implications for aging, mental health and forensic researchers, clinicians and public health services, and calls attention to advancing the understanding of familial factors in externalizing behaviors and aging.

## Background and Objectives

Externalizing behavioral spectrum compromises a wide range of behaviors characterized by general behavioral disinhibition that violate social norms and/or are harmful to others like aggression, violence, substance use, theft, or property destruction (Kauten & Barry, 2020; Vaughn et al., 2014). Previous research has indicated that individuals who show externalizing behaviors such as criminal behavior and substance abuse may develop antisocial lifestyles and risk-taking behaviors which may increase the rates of mental and physical illness, and premature mortality in adulthood (Hamdi & Iacono, 2014; Kendler et al., 2018; Moffitt, 2018). However, most of the studies investigating the influence of externalizing problems (e.g., criminal behavior and substance abuse) in the development of negative health outcomes have followed individuals until mid-adulthood excluding from their studies cohorts of older individuals.

Dementia refers to a group of disorders, generally major neurodegenerative disorders, such as Alzheimer’s disease (AD) or vascular dementia, characterized by a decline in cognitive abilities, behavioral changes, and impairment in daily functionality (WHO, 2022). Dementia is one of the major causes of disability among older adults and it represents a public health problem worldwide (WHO, 2022). The short- and long-term neurodegenerative, cognitive, and functional deficits associated with some externalizing problems such as drug abuse (i.e., severe or chronic alcoholism) are well documented (Cheng et al., 2017; Stavro et al., 2013). Nevertheless, very few studies have investigated the association of other externalizing problems, like violent and nonviolent criminal behavior along the life course, with the development of different types of dementia in aging. The limited available evidence suggests an increased risk of cognitive decline and dementia in older individuals with criminal background (Ahalt et al., 2018; Forsyth et al., 2020), but research on this topic is still scarce. Considering that the global population is getting older and that the number of older individuals aging with drug abuse or inside forensic and penal institutions is rising (International Narcotics Control Board, 2021; Kriminalvård, 2020; U.S. Bureau of Justice, 2016), further research exploring associations between externalizing behaviors and dementia outcomes are warranted.

Several potential processes may explain an association between externalizing problems and dementia. First, because of their lifestyle, individuals with externalizing behaviors may develop physical and psychiatric health problems, as well as a variety of adverse psychosocial circumstances (Hamdi & Iacono, 2014; Kendler et al., 2018; Langevin et al., 2022; Moffitt, 2018), which constitute risk factors for dementia and could explain an increase in the risk of these individuals for accelerated cognitive decline and dementia in aging. Indeed, a recent study using data from the Dunedin Multidisciplinary Health and Development database (Langevin et al., 2022), has shown that individuals with a history of antisocial behavior experienced accelerated biological aging. Accelerated aging in these individuals was driven by the accumulation of adverse lifestyle and psychosocial factors. Although accelerated biological aging may increase the risk of dementia, no specific associations between antisocial behavior and dementia were explored in this study. In this line, a longitudinal register-based cohort study using Swedish registers (Solares et al., 2023) showed that individuals with a criminal background were at higher risk for dementia and MCI. The study showed that the risk for both outcomes was partially explained by a combination of negative health and psychosocial life-course factors, and especially by a history of mental health problems. However, this study did not explore how other factors such as biological, genetic, and familial predispositions may influence the associations. Thus, further research is needed to understand the role of familial-related factors on the associations between externalizing behaviors and different types of dementia.

Second, family-based studies on the development of antisocial behavior have reported a strong genetic and environmental risk for violent crime and other externalizing behaviors such as alcohol abuse (Frisell et al., 2011; Kendler et al., 2018). The onset of some types of dementia, such as AD, is also influenced by many health and lifestyle factors, such as cardiovascular problems, depression, and low educational level, as well as by familial and genetic risk factors. Genetic risk factors include being a carrier of E4 allele of the apolipoprotein E gene and a still-growing number of genetic variants, that influence the pathogenesis of the disease and increase the risk of developing dementia in late adulthood and aging (Licher et al., 2019; Magnin, 2021). Given that externalizing behaviors and dementia seem to be heritable and complex conditions (Kendler et al. 2016; Licher et al., 2019), together with previous research suggesting a higher risk of dementia development in individuals with criminal and other externalizing problems, it is feasible to consider that externalizing behaviors and dementia may share familial risks. Hence, in the present study, we aim to investigate whether externalizing behaviors, AD, and other dementias co-aggregate in families. So far, no genome-wide association study (GWAS) has explored the genetic associations between criminal or other externalizing problems and dementia. Furthermore, up until now, there are no longitudinal studies available, probably because such studies would require access to large-scale longitudinal data with very long follow-up time. Nevertheless, multigeneration cohorts offer the opportunity to develop study designs where relatives with different familial relatedness (e.g., siblings, offspring, parents, grandparents) can be followed across time which allows for the tracking of different conditions across generations, which is needed to understand the role of intergenerational transmission as well as the influence of genetic and family-wide environmental factors (Kretschmer, 2021). Thus, in the present study, we performed a longitudinal multigeneration study to explore the association between externalizing behaviors (i.e., criminal behavior, substance abuse) and AD and other dementias, across generations (i.e., parents, grandparents, and uncles/aunts), using data from the linkage of Swedish national registers.

## Research Design and Methods

### Data Sources

Several Swedish population-based registers were linked using unique personal identification numbers (Ludvigsson et al., 2016). We extracted data from the following Swedish registers: (a) *The Medical Birth Register* (*MBR*) includes information on pre-, peri- and post-natal factors of almost all births in Sweden since 1973; (b) *Total Population Register* (*TPR*) includes information on all individuals born in Sweden after 1932 and who were alive in 1968 or later. TPR contains the following demographics: date of birth, sex, country of birth, migration, and date of death (Ludvigsson et al., 2016); (c) *The MultiGeneration Register* (*MGR*) contains information on biological parents of all individuals born after 1932 and who are alive and living in Sweden since 1961; (d) *The National Patient Register* (*NPR*) provides diagnoses of all in-patients from 1987 and all out-patient visits from 2001. Diagnoses are coded using the International Classification of Diseases (ICD) versions 7/8/9/10 (Ludvigsson et al., 2011); (e) *The Cause of Death Register* (*CDR*) contains dates and causes of all deaths since 1952 (Brooke et al., 2017); (f) *The Prescribed drug register* (*PDR*) includes information on all prescribed medications dispensed in Sweden since July 2005 (Wettermark et al., 2007). Medication identity is defined using the Anatomical Therapeutic Chemical (ATC) classification; (g) *The Migration Register* (*MR*) provides records of in and out migrations from Sweden; and (h) *The National Crime Register* (*NCR*) contains information on all criminal convictions, including noncustodial sentences and fines, in Swedish law courts since 1973 (Chang et al., 2015).

### Study Population

We selected a main cohort of offspring for which we obtained information about externalizing behaviors. We collected information on individuals older than 15 years old and living in Sweden between 1988 and 2013. Individuals in the main cohort were born between the years 1973 and 1997. We excluded stillbirths, individuals missing important demographic information, and individuals who died or migrated before their 15th birthday, by linking to the TPR, MBR, MR, and CDR. Individuals from the offspring cohort (index persons) were linked to their biological relatives—parents, grandparents, and uncles/aunts—using the MBR and MGR. All relatives were followed from the date they turned 50 years of age to the onset of dementia, date of first migration, date of death, or December 31, 2013 (end of study follow-up) whichever came first. Therefore, we created three cohorts of relatives that represent different levels of familial relatedness with index persons: parents (50%), grandparents (25%), and uncles/aunts (25%; Zhang et al., 2021). Finally, for each parent of each index person, we selected one uncle/aunt who had at least one child and whose birth date was nearest to that of the parents of index persons (Zhang et al., 2021). We decided on this selection in order to guarantee that parents and uncles/aunts cohorts were comparable.

### Measures of Externalizing Behavior

Three measures were used to cover different aspects of the externalizing spectrum (a) Nonviolent crime: to have a criminal record of convictions for nonviolent crimes; (b) Violent crime: to have a criminal record of convictions for violent crimes. Based on previous research, violent crime was defined as murder, homicide, assault, robbery, arson, any sexual offense (rape, sexual coercion, child molestation, indecent exposure, or sexual harassment), illegal threats, or intimidation (Frisell et al., 2011; Latvala et al., 2015; Solares et al., 2023). Nonviolent crime was defined as any other crime than violent crimes (e.g., fraud, vandalism, and theft); (c) substance use disorders (SUD): to have a recorded diagnosis of SUD. We used information from the NCR to identify criminal conviction records and from the NPR to identify SUD diagnoses among index persons (see Supplementary Table 4).

### Measures of AD and Any Dementia

We used information from the NPR and the CDR to collect Alzheimer’s disease and any dementia (including AD) diagnoses among relatives of index persons. (See Supplementary Table 1 for the ICD codes and the drug prescriptions codes). As dementia is a progressive disorder and initial symptoms tend to be silent, the time when a dementia case is detected and entered the Swedish registers may differ from the true onset of clinically diagnosed dementia (Rizzuto et al., 2018). Therefore, based on previous research (Jin et al., 2004; Rizzuto et al., 2018; Zhang et al., 2021) we estimated the time of disease onset as three years before the first diagnosis recorded in the NPR or five years before a cause of death established in the CDR, whichever came first. Finally, for the sensitivity analysis, we further identified the diagnosis of AD using medication prescription for Alzheimer’s disease from the PDR.

### Statistical Analyses

In the main analyses, separate Cox proportional hazards models were performed to examine the associations between each measure of the externalizing behaviors, AD, and any dementia in each of the relative cohorts (parents, grandparents, and uncles/aunts). Hazard ratios (HRs) were estimated with 95% confidence intervals (CIs), using the attained age of the relatives as the underlying time scale. To account for the nonindependence of data due to the repeat of individuals in index person-relative pairs, cluster-robust estimations were used. The risk of having dementia in relatives of individuals with externalizing behaviors was compared with the risk in relatives of individuals without externalizing behaviors. In the adjusted models, HRs were adjusted for the birth year of index person, birth year of relatives, sex of index person, and the sex of relatives. The analysis was further stratified by sex of relatives. Finally, HRs were estimated for early-onset dementia (onset before 65 years) and late-onset dementia (onset after 65 years old) in each relative cohort.

Sensitivity analyses were performed to explore the robustness of the results regarding the contribution of common familial risk factors for externalizing behavior and AD and any dementia. Thus, we collected data of records for the three measures of externalizing behavioral spectrum along relatives’ life and, then we adjusted our analyses by adding as a covariate the presence of the measures of externalizing behaviors for each of the three relative cohorts. Finally, we aimed to improve the coverage of AD diagnosis by including prescriptions for AD medication to identify AD cases. Data management was performed using SAS version 9.4 (SAS Institute, Inc.), and R version 4.1.0 was used for data analyses.

## Results

**Figure 1.**
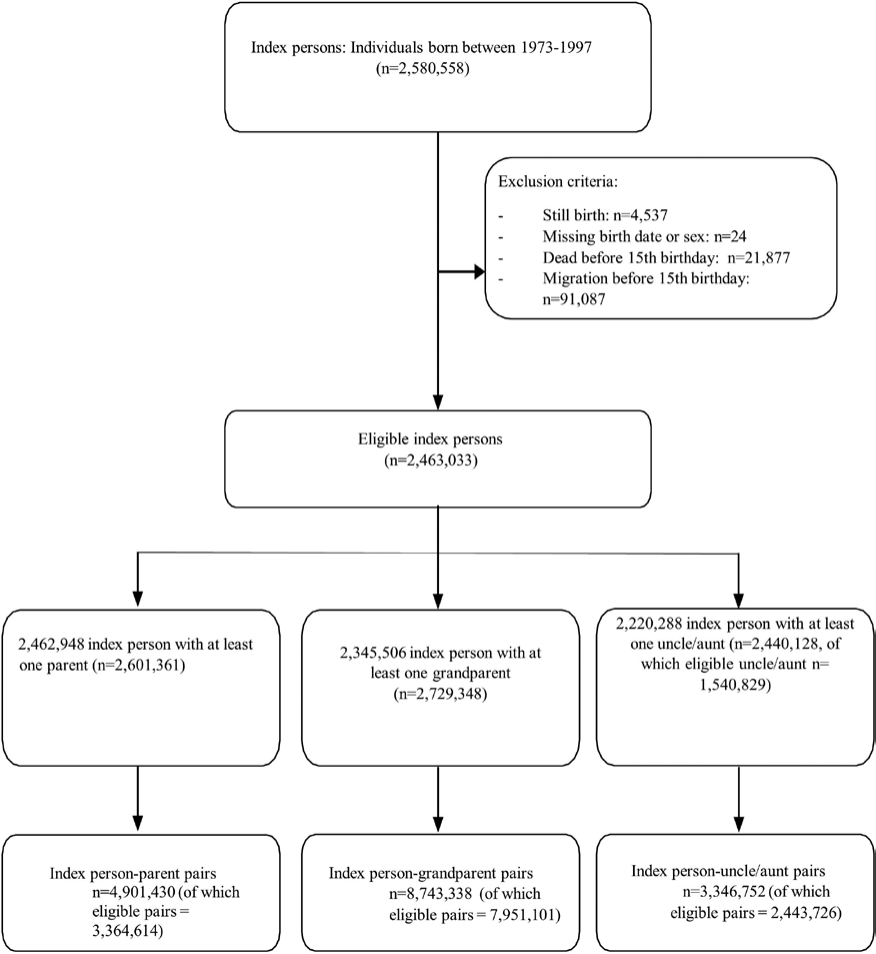
Flowchart of the inclusion of index persons and their biological relatives.

A total of 2,580,558 individuals born between 1973 and 1997 were identified from the Medical Birth Register. After applying the exclusion criteria there were 2,463,033 individuals who were eligible index persons. Index persons were linked to their biological relatives to create three study cohorts (i.e., parents, grandparents, and uncles/aunts) containing 2,601,361 parents, 2,729,348 grandparents and 1,540,829 uncles/aunts, which created 4,901,430 index person-parent pairs, 8,743,338 index person-grandparent pairs and 3,346,752 index person-uncle/aunt pairs (see Figure 1). Among the index persons, 112,526 (4.57%) had a diagnosis of SUD (64,083 were men), 293,878 (11.93%) were convicted of nonviolent crimes (207,776 were men) and 86,905 (3.53%) were convicted for violent crimes (73,835 were men). The number of dementia cases was 11,811 (0.45%) for parents, 253,602 (9.29%) for grandparents, and 6,980 (0.45%) for uncles/aunts (see Table 1). Parents were followed for a median of 9.96 years, grandparents for a median of 27.37 years, and uncles/aunts for a median of 10.46 years.

**Table 1.** Descriptive Characteristics of Index Persons and the Three Relative Cohorts

Type of individual	Variable	Overall	Women	Men
Index person	*N* of index person	2,463,033	1,198,443	1,264,590
	Age, Median (IQR)^a^	28 (22–35)	28 (22–35)	28 (22–35)
	Nonviolent crime, n (%)	293,878 (11.93)	86,102 (3.5)	207,776 (8.44)
	Violent crime, n (%)	86,905 (3.53)	13,070 (0.53)	73,835(3)
	SUD, *N* (%)	112,526 (4.57)	48,443 (1.97)	64,083 (2.60)
Parents	*N* of parents	2,601,361	1,303,234	1,298,127
	Age, Median (IQR)^a^	58 (51–65)	57 (50–64)	59 (52–66)
	Alzheimer Disease, *n* (%)	9,307 (0.36)	3,348(0.13)	5,959(0.23)
	Any dementia, *n* (%)	11,811 (0.45)	4,024 (0.15)	7,787 (0.30)
	Onset age of Alzheimer’s disease, Median (IQR)^b^	65 (59–71)	62 (57–67)	67 (61–73)
	Onset age of Any dementia, Median (IQR)^b^	65 (59–71)	62 (57–67)	66 (60–72)
Grandparents	*N* of grandparents	2,729,348	1,284,719	1,344,629
	Age, Median (IQR)^a^	76 (70–84)	76 (70–83)	77 (71–84)
	Alzheimer Disease, *n* (%)	221,056 (8.10)	133,574 (4.89)	87,482 (3.21)
	Any dementia, *n* (%)	253,602 (9.29)	149,676 (5.48)	103,926 (3.81)
	Onset age of Alzheimer’s disease, median (IQR)^b^	79 (74–84)	80 (75–84)	78 (73–83)
	Onset age of Any dementia, median (IQR)^b^	79 (74–83)	80 (75–84)	78 (73–82)
Uncles/aunts	*N* of uncles/aunts	1,540,829	784,895	755,934
	Age, Median (IQR)^a^	58 (50–65)	58 (50–65)	58 (50–65)
	Alzheimer Disease, *n* (%)	5,599 (0.36)	2,965 (0.19)	2,634 (0.17)
	Any dementia, *n* (%)	6,980 (0.45)	3,487 (0.23)	3,493 (0.23)
	Onset age of Alzheimer’s disease, Median (IQR)^b^	65 (60–69)	65 (59–69)	65 (60–69)
	Onset age of Any dementia, Median (IQR)^b^	65 (59–69)	65 (59–69)	65 (59–69)

*Notes*: IQR = interquartile range; SUD = substance use disorder.

^a^The age of individuals by the end of the study.

^b^Time of disease onset was estimated as 3 years before the first diagnosis, or 5 years before death, whichever comes first.

Parents of index persons with externalizing behaviors had an increased risk for AD compared with parents of index persons without externalizing problems (nonviolent crime: HR = 1.16 [95% CI: 1.10–1.22]; violent crime: HR = 1.32 [95% CI: 1.19–1.45]; SUD: HR = 1.28 [95% CI: 1.17-1.40], see Table 2). When decreasing the familial relatedness, the associations between nonviolent crime and violent crime with AD were attenuated for both grandparents (nonviolent crime: HR = 1.05 [95% CI: 1.04-1.06]; violent crime: HR = 1.07 [95% CI: 1.05–1.09]) and uncles/aunt (see Table 2). The associations between SUD and AD were also attenuated for both relative cohorts but were not statistically significant for uncles/aunts (SUD: grandparents 1.07 [95% CI: 1.06, 1.09]; uncles/aunts 1.05 [95% CI: 0.94, 1.18]). We observed a similar pattern of results for the associations with any dementia, where parents of index persons with externalizing problems showed an increased risk for any dementia (nonviolent crime: HR = 1.17 [95% CI: 1.11–1.22]; violent crime: HR = 1.39 [95% CI: 1.27–1.52]; SUD: HR = 1.33 [95% CI: 1.23–1.43]), which was attenuated in grandparents and uncles/aunts (see Table 3 for a complete report of the results).

**Table 2.** Associations Between Externalizing Behaviors and Alzheimer’s Disease in Three Relative Cohorts

Relative of index Persons	No criminal background		Nonviolent crime				Violent crime				No SUD background		SUD			
	*N* events	Person–years	*N* events	Person-years	Crude HRs (95% CI)	Adjusted HRs (95% CI)	*N* events	Person-years	Crude HRs (95% CI)	Adjusted HRs (95% CI)	*N* events	Person-years	*N* events	Person-years	Crude HRs (95% CI)	Adjusted HRs (95% CI)
Parents	9,648	29,488,460	1,818	4,572,893	1.17 (1.11, 1.23)	1.16 (1.10, 1.22)	451	1,098,609	1.30 (1.18, 1.43)	1.32 (1.19,1.45)	11,359	33,751,194	558	1,408,768	1.27 (1.16, 1.38)	1.28 (1.17,1.40)
Mother	3,398	13,095,836	646	2,064,439	1.18 (1.08, 1.28)	1.18 (1.08,1.30)	159	494,946	1.29 (1.10, 1.51)	1.35 (1.15, 1.60)	3,989	15,031,335	214	623,886	1.39 (1.21, 1.59)	1.45 (1.26, 1.67)
Father	6,250	16,392,624	1,172	2,508,453	1.16 (1.09, 1.24)	1.15 (1.08, 1.23)	292	603,663	1.31 (1.16, 1.47)	1.30 (1.15, 1.48)	7,370	18,719,858	344	784,881	1.19 (1.07, 1.34)	1.20 (1.07, 1.33)
Grandparents	596,810	182,755,836	85,233	24,605,108	1.05 (1.05, 1.06)	1.05 (1.04, 1.06)	20,703	6,389,812	1.08 (1.06, 1.09)	1.07 (1.05, 1.09)	673,668	204,930,738	29,078	8,820,018	1.08 (1.07, 1.09)	1.07 (1.06, 1.09)
Grandmother	350,018	94,874,543	50,965	12,913,714	1.05 (1.05, 1.06)	1.05 (1.04, 1.06)	12,388	3,361,761	1.09 (1.07, 1.11)	1.09 (1.06, 1.11)	396,274	106,532,557	17,097	4,617,462	1.08 (1.06, 1.09)	1.08 (1.06, 1.10)
Grandfather	246,792	87,881,293	34,268	11,691,394	1.05 (1.04, 1.06)	1.05 (1.04, 1.06)	8,315	3,028,050	1.06 (1.03, 1.08)	1.05 (1.02, 1.07)	277,394	98,398,181	11,981	4,202,556	1.08 (1.06, 1.1)	1.07 (1.05, 1.09)
Uncles/aunts	7,719	22,636,946	1,292	3,398,660	1.08 (1.02, 1.15)	1.07 (1.00, 1.13)	319	826,863	1.18 (1.04, 1.32)	1.19 (1.06, 1.34)	8,978	25,797,252	352	1,065,219	1.02 (0.91, 1.14)	1.05 (0.94, 1.18)
Aunt	4,101	11,522,197	694	1,745,453	1.09 (1.00, 1.18)	1.07 (0.98, 1.16)	162	427,783	1.10 (0.93, 1.30)	1.11 (0.94, 1.32)	4,782	13,148,161	175	547,273	0.94 (0.81, 1.09)	0.96 (0.82, 1.14)
Uncle	3,618	11,114,748	598	1,653,207	1.08 (0.99, 1.18)	1.06 (0.97, 1.17)	157	399,080	1.26 (1.07, 1.50)	1.29 (1.08, 1.53)	4,196	12,649,090	177	517,945	1.11 (0.95, 1.29)	1.15 (0.98, 1.35)

*Notes*: CI = Confidence interval; HRs = Hazard Ratios; SUD = substance use disorder.

*N* of events and person-years are shown for index person-relative pair (not individuals); Adjusted Hazard Ratios (HRs) were derived from cox proportional models adjusted by birth year of index person, birth year of relatives, sex of index person, and sex of relatives.

**Table 3.** Associations Between Externalizing Behaviors and any Dementia in Three Relative Cohorts

Relative of index Persons	No criminal background		Nonviolent crime				Violent crime				No SUD background		SUD			
	*N* events	Person-years	*N* events	Person-years	Crude HRs (95% CI)	Adjusted HRs (95% CI)	*N* events	Person-years	Crude HRs (95% CI)	Adjusted HRs (95% CI)	*N* events	Person-years	*N* events	Person-years	Crude HRs (95% CI)	Adjusted HRs (95% CI)
Parents	12,363	29,471,047	2,342	4,569,380	1.18 (1.12, 1.23)	1.17 (1.11,1.22)	610	1,097,568	1.38 (1.27, 1.49)	1.39 (1.27, 1.52)	14,572	33,730,439	743	1,407,557	1.31 (1.22, 1.41)	1.33 (1.23, 1.43)
Mother	4,098	13,091,106	800	2,063,391	1.21 (1.12, 1.31)	1.22 (1.12, 1.32)	191	494,722	1.28 (1.11, 1.48)	1.35 (1.16, 1.57)	4,839	15,025,581	250	623,639	1.33 (1.17, 1.51)	1.39 (1.22, 1.59)
Father	8,265	16,379,940	1,542	2,505,988	1.16 (1.10, 1.22)	1.15 (1.08,1.21)	419	602,846	1.42 (1.29, 1.57)	1.42 (1.27,1.57)	9,733	18,704,857	493	783,917	1.29 (1.18, 1.42)	1.30 (1.18, 1.43)
Grandparents	686,601	182,173,073	97,879	24,522,018	1.05 (1.05, 1.06)	1.05 (1.04, 1.06)	23,948	6,368,423	1.08 (1.07, 1.09)	1.07 (1.06, 1.09)	774,893	204,272,794	33,535	8,790,720	1.08 (1.07, 1.09)	1.07 (1.06, 1.09)
Grandmother	392,833	94,580,007	57,085	12,871,166	1.05 (1.04, 1.06)	1.05 (1.04, 1.06)	13,957	3,350,555	1.09 (1.07, 1.11)	1.09 (1.07, 1.11)	444,702	106,198,841	19,173	4,602,888	1.08 (1.06, 1.09)	1.08 (1.06, 1.10)
Grandfather	293,768	87,593,065	40,794	11,650,851	1.05 (1.04, 1.06)	1.05 (1.04, 1.07)	9,991	3,017,868	1.07 (1.04, 1.09)	1.06 (1.03, 1.08)	330,191	98,073,953	14,362	4,187,832	1.09 (1.07, 1.1)	1.07 (1.05, 1.09)
Uncles/aunts	9,739	22,623,454	1,640	3,396,283	1.09 (1.03, 1.15)	1.08 (1.02, 1.14)	404	826,333	1.18 (1.06, 1.31)	1.20 (1.08, 1.33)	11,343	25,781,420	440	1,064,652	1.00 (0.91, 1.1)	1.04 (0.94, 1.15)
Aunt	4,849	11,516,863	842	1,744,441	1.12 (1.04, 1.2)	1.10 (1.02, 1.19)	196	427,549	1.13 (0.98, 1.3)	1.14 (0.98, 1.33)	5,676	13,141,829	211	547,024	0.95 (0.83, 1.09)	0.98 (0.84, 1.13)
Uncle	4,890	11,106,591	798	1,651,842	1.07 (0.99, 1.15)	1.05 (0.97, 1.14)	208	398,784	1.23 (1.07, 1.42)	1.25 (1.08, 1.45)	5,667	12,639,590	229	517,627	1.06 (0.93, 1.21)	1.09 (0.96, 1.26)

*Notes*: CI = Confidence interval; HRs = Hazard Ratios; SUD = substance use disorder.

*N* of events and person-years are shown for index person-relative pair (not individuals); adjusted Hazard Ratios (HRs) were derived from cox proportional models adjusted by birth year of index person, birth year of relatives, sex of index person, and sex of relatives.

The estimates that were stratified by sex of the relatives showed that female relatives (i.e., mothers and grandmothers) of index persons with externalizing problems had similar risk for AD as male relatives, even though a higher but nonstatistically significant risk was observed in females compared with males for SUD (mothers: Nonviolent crime: HR = 1.18 [95% CI: 1.09–1.29]; violent crime: HR = 1.35 [95% CI: 1.15–1.59]; SUD: HR = 1.45 [95% CI: 1.26, 1.66]; fathers: nonviolent crime: HR = 1.15 [95% CI: 1.08–1.23]; violent crime: HR = 1.30 [95% CI: 1.16–1.47]; SUD: HR = 1.20 [95% CI: 1.07, 1.33]). The associations with any dementia followed the same pattern of results (for a complete report of results see Tables 2 and 3). In general, when considering age of onset for AD and any dementia, relatives of index persons with externalizing problems tend to show a higher risk for having early-onset AD and any dementia than a late-onset of the diseases. However, differences between early- and late-onset diseases was only statistically significant for the grandparents cohort: Early-onset dementia (nonviolent crime: HR = 1.16, 95% CI: 1.11–1.20; violent crime: HR = 1.17, 95% CI: 1.10–1.25; SUD: HR = 1.16, 95% CI: 1.10, 1.22), Late-onset any dementia (nonviolent crime: HR = 1.04, 95% CI: 1.04-1.05; violent crime: HR = 1.07, 95% CI: 1.05–1.08; SUD: HR = 1.07, 95% CI: 1.06, 1.08; see Table 4 for a complete report of results for parents, grandparents and uncles/aunts relative cohorts).

**Table 4.** Associations Between Externalizing Behaviors and Alzheimer’s Disease and Any Dementia Stratified by Onset Age of Alzheimer’s Disease or Any Dementia

Relative of index persons	Age at onset	No criminal background	Nonviolent crime		Violent crime		No SUD background	SUD	
		*N* events	*N* events	Adjusted HRs (95% CI)	*N* events	Adjusted HRs (95% CI)	*N* events	*N* events	Adjusted HRs (95% CI)
Alzheimer’s disease									
Parents	Early-onset	5,251	1,001	1.20 (1.12, 1.29)	237	1.24 (1.08, 1.43)	6,163	326	1.33 (1.18, 1.49)
	Late-onset	4,397	817	1.09 (1.01, 1.18)	214	1.35 (1.16, 1.56)	5,196	232	1.20 (1.04, 1.38)
Grandparents	Early-onset	23,211	3,500	1.16 (1.11, 1.20)	975	1.15 (1.07, 1.23)	26,285	1,401	1.17 (1.10, 1.24)
	Late-onset	573,599	81,733	1.05 (1.04, 1.05)	19,728	1.06 (1.05, 1.08)	647,383	27,677	1.07 (1.06, 1.08)
Uncles/aunts	Early-onset	4,150	693	1.08 (0.99, 1.17)	183	1.21 (1.03, 1.42)	4,821	295	1.06 (0.91, 1.24)
	Late-onset	3,569	599	1.04 (0.95, 1.13)	136	1.15 (0.96, 1.38)	4,157	147	1.02 (0.86, 1.21)
Any dementia									
Parents	Early-onset	6,841	1,337	1.24 (1.16, 1.32)	340	1.37 (1.23, 1.53)	8,073	445	1.38 (1.25, 1.53)
	Late-onset	5,522	1,005	1.06 (0.99, 1.14)	270	1.35 (1.19, 1.53)	6,499	298	1.23 (1.09, 1.39)
Grandparents	Early-onset	29,221	4,382	1.16 (1.11, 1.20)	1,259	1.17 (1.10, 1.25)	33,100	1,762	1.16 (1.10, 1.22)
	Late-onset	657,380	93,497	1.04 (1.04, 1.05)	22,689	1.07 (1.05, 1.08)	741,793	31,773	1.07 (1.06, 1.08)
Uncles/aunts	Early-onset	5,447	900	1.07 (0.99, 1.15)	233	1.17 (1.03, 1.35)	6,317	263	1.04 (0.91, 1.19)
	Late-onset	4,292	740	1.07 (0.98, 1.16)	171	1.21 (1.03, 1.42)	5,026	177	1.02 (0.87, 1.19)

*Notes*: CI = confidence interval; HRs = Hazard Ratios; SUD = substance use disorder.

*N* of events are shown for index person-relative pair (not individuals); adjusted Hazard Ratios (HRs) were derived from cox proportional models adjusted by birth year of index person, birth year of relatives and sex of index person.

### Sensitivity Analyses

To explore the robustness of the results regarding the contribution of common familial risk factors to externalizing behaviors and dementia, we further adjusted the analysis for externalizing problems in relatives. The results adjusting for externalizing problems in relatives were similar to the main results where the risk of having any dementia for relatives of index persons with externalizing behaviors was attenuated in comparison to the main results but still significant for all the analyses, except for the associations with SUD in the uncle/aunt cohort (see Supplementary Table 2). The inclusion of prescriptions for AD medication for case identification also yielded results consistent with the main analysis for the associations across the three relative cohorts (see Supplementary Table 2).

## Discussion and Implications

In this population-based registry study, we aimed to explore familial associations between externalizing behaviors and dementia across generations. We investigated whether relatives of individuals with externalizing problems, specifically those with a history of violent criminal behavior, nonviolent criminal behavior, or SUD, had an increased risk for AD or any dementia. We found that relatives of individuals with externalizing problems showed a higher risk of AD and any dementia compared with relatives of individuals without. The associations were attenuated as a function of reduced familial relatedness, that is associations were stronger for parents of index-persons than for grandparents or uncles/aunts. In addition, we found that relatives of individuals with externalizing problems compared with relatives of individuals without showed an increased risk of having both early-onset and late-onset dementia.

The finding of familial coaggregation between externalizing problems with AD and any dementia is in line with previous evidence showing an elevated risk of dementia onset in individuals with SUD (Richmond-Rakerd et al., 2022), but also provides new and important information about the association between criminal behavior along the life course and dementia onset in aging. There may be several potential explanations for the observed familial coaggregation of externalizing behaviors with AD and any dementia. Therefore, we suggested and described four potential underlying mechanisms contributing to the observed familial co-aggregation between externalizing behaviors in the index person and dementia in his/her relatives. A Directed Acyclic Graph (DAG) illustrating the relationship between conditions can be found in online supplementary material (see Supplementary Figure 1, path a–d). First, there may be familial risk factors, both genetic and family-wide environmental factors, that may influence both conditions (see path a, Supplementary Figure 1). Previous studies have suggested that a frequently occurring single-nucleotide polymorphism (SNP) of the KIBRA gene (rs17070145), may be implicated in cognitive performance, especially memory performance, and may constitute a modest risk gene for AD and other dementias such as vascular dementia, but also SUD (Bauer et al., 2012; Kawai et al., 2015; Mazzeo et al., 2019; Wang et al., 2021). In addition, a recent study (Ramos et al., 2022) suggests that alcohol use and neurodegenerative disorders such as AD may have overlapping genetic risk factors that involve the activation of the innate immune system. To the best of our knowledge, no previous study has explored common genetic variants between violent or criminal behavior and AD or any other dementia. Previous studies have reported that dysfunction in cortical and subcortical brain networks including orbitofrontal cortices, amygdala, and somatosensory and insular brain areas may underly impulsivity, aggression, antisocial personality traits, and violent behavior (Carlisi et al., 2020; Talaslahti et al., 2021), as well as specific types of neurodegenerative disorders such as the behavioral-variant of frontotemporal dementia (Bv-FTD; Cipriani et al., 2020; Talaslahti et al., 2021). Thus, it is possible that genetic variants involved in neural degeneration and dysfunctions of these brain areas may influence the development of criminal and violent behavior, but also the development of at least some types of dementia. Further research is needed to explore whether these two conditions share common genetic variants. Factors related to family-wide environment may also play a role in the association between externalizing behaviors and dementia (Ridge et al., 2016; Zhang et al., 2021). For instance, evidence exists that socioeconomic status may influence the development of SUD (Patrick et al., 2012), criminal behavior (Farrington et al., 2017), and dementia (Marden et al., 2017). In addition, these shared familial risk factors may not just indicate between-individual associations across generations (e.g., between index person and parents), but also individual-level associations between externalizing behaviors and AD or any dementia.

A second potential explanation for the observed coaggregation of externalizing behaviors with AD or any dementia across generations may be the influence of genetic and environmental risk factors unique to externalizing problems (see path b, Supplementary Figure 1). Indeed, family-based studies on the development of antisocial and criminal behavior have reported strong familial risk for violent crime and other externalizing behaviors such as alcohol abuse (Frisell et al., 2011; Kendler, Ohlsson, Sundquist & Sundquist, 2018). Thus, it may be that these familial risk factors affect the development of externalizing problems in both the index persons and their relatives and that the risk of having dementia in relatives is mediated by the adverse health and psychosocial outcomes triggered by their externalizing behaviors (Zhang et al., 2021). For instance, individuals with SUD and criminal behaviors may show poor healthy lifestyle habits, an increased risk of acquired brain injuries, or developing cardiovascular diseases such as hypertension or stroke, which are well-established risk factors for the development of dementia (Fillit et al., 2008; Norton et al., 2014). Moreover, this explanation may also lead to the assumption of an individual-level association between both conditions. An individual-level association between SUD and dementia has previously been shown in several studies exploring cognitive decline associated with drug abuse (Ramey & Regier, 2019) and alcohol-related dementia (Sachdeva et al., 2016). Studies investigating the risk of AD and other dementia in individuals with criminal backgrounds are still scare. Nevertheless, older adults with criminal background appear to be more prone to dementia than the general population (Cipriani et al., 2017) because they are highly exposed to risk factors for dementia along their life course. Furthermore, the prevalence of any dementia is estimated to be around 7%–8% among older offenders (Forsyth et al., 2020; Solares et al., 2020). Thus, our study provides new insights into the associations between criminal behavior and dementia among individuals from the same family, and it also sheds light on potential underlying mechanisms for the associations between externalizing problems and AD and other dementias.

Familial coaggregation of externalizing behaviors and dementia could also be related through the effect of externalizing behaviors in the relatives directly influencing their own risk for AD or any other dementia, as well as the risk of having externalizing behaviors in the index person (Zhang et al., 2021; see path c, Supplementary Figure 1). Parents with SUD or criminal behavior may create an unstable upbringing and exhibit low parental concern and skills which have been reported as risk factors for criminal and externalizing problems during adolescence and early adulthood (Corovic et al., 2017). Nevertheless, after a sensitivity analysis adjusting the estimates for externalizing problems in the relatives, the associations remained significant. These results show that the presence of externalizing problems in the relatives is not the main reason for the observed association across generations.

A last potential mechanism might implicate a direct effect of the externalizing behaviors in the index person on the development of dementia in the relatives (see path d, Supplementary Figure 1). This may be explained by the possibility that parents of individuals who engage in SUD or criminal behaviors may experience depression and psychological distress which may increase their risk of developing dementia. However, this mechanism may not help to understand why the associations are maintained when the familial relatedness is reduced (Zhang et al., 2021).

We also found that relatives of individuals with externalizing problems compared with relatives of individuals without showed an increased risk of having both early-onset and late-onset dementia. The associations were stronger with early-onset dementia than with late-onset dementia, although the difference was only statistically significant for grandparents. Early-onset dementia is highly genetically determined, in most cases the inheritance is consistent with autosomal dominant transmission, and genetic analyses have identified mutations in three genes coding for the amyloid precursor protein (APP) and the presenilins 1 and 2 (PSEN1 And PSEN2) involved in early onset dementia (Cacace et al., 2016). Moreover, GWAS has shown that late-onset dementia is influenced by a diverse variation of genes that mainly affect three biological pathways: the immune system, lipid metabolism, and synaptic dysfunction/cell membrane processes (Cacace et al., 2016). The difference in the inheritance of early- and late-onset dementia may contribute to the stronger association with early-onset AD and other dementias that we observed in relatives of index persons with externalizing behaviors. However, the difference in the HRs of early onset and late-onset AD or any dementia were only statistically significant for grandparents. The median age of parents of index persons at the end of the follow-up was below 60 years old and they were followed for a shorter time period than grandparents (i.e., 9.96 vs 27.37 years of follow-up). Considering that individuals with early-onset dementia are often misdiagnosed with other psychiatric disorders (McMurtray., Clark, Christine, & Mendez, 2006), it may be the case that the follow-up time-period of parents in the present study did not allow us to capture those early-onset dementia diagnoses that are established after a first misdiagnosis of a psychiatric disorder. Further research is needed to explore the association between externalizing problems and early-onset dementia and to investigate whether the associations are driven by familial risk factors.

The use of a multigenerational design where data on externalizing behaviors and dementia cases were collected separately in the index person and relatives provided several advantages such as ruling out reverse causation as a potential explanation for the association. This design is particularly useful to explore associations where individual longitudinal data is required but is not available. For instance, when aiming for exploring associations between conditions that may occur early in life or along the life course, with conditions that occur in late adulthood. Nevertheless, this design has several limitations. First, we could not include data on specific genes, biomarkers, or other life-course risk factors to explore the associations since data were not available in the registers. However, the use of a multigenerational design provided general information about familial-related contributions to the associations, and it allowed us to build up lifespan associations indirectly. Future studies with access to specific biological and psychosocial data may explore, in greater detail, the role of genetics, biomarkers, or psychosocial factors that may explain the associations between externalizing behaviors and dementia found in the present study. Second, we lacked statistical power to explore the associations between specific types of SUD with AD and any dementia. Similarly, we were not able to explore the associations with different types of dementia (e.g., vascular dementia, frontotemporal dementia, and Lewy body dementia). Individuals with externalizing behaviors frequently report behaviors such as aggression, agitation, delusions, hallucinations, or disinhibition which are also common noncognitive neuropsychiatric symptoms (NPS) of some types of dementia (Kales et al., 2014). Thus, it may be the case that our results of an association between externalizing problems and any dementia are driven by specific subtypes of dementia such as frontotemporal, Lewy body, or vascular dementia. Further studies are needed to explore whether individuals with externalizing problems are more prone to develop specific types of dementia and to investigate if the underlying neural dysfunctions of NPS are also common dysfunctions between conditions. Third, due to the study design and a lack of available information about the diagnosis of neurodevelopmental disorders mainly for the relative cohorts, we were not able to explore how neurodevelopmental conditions may potentially influence the hazards of a familial co-aggregation of externalizing behaviors and dementia. Previous research has already described how different neurocognitive deficits in childhood and adolescence, such as neurodevelopmental problems, prefrontal deficit dysfunction or low general cognitive ability are risk factors for psychiatric and neurocognitive problems later in life. Such factors have also been related to an increased risk for criminal and other externalizing behaviors such as substance abuse (Lundström et al., 2014). Future studies may investigate the role of neurodevelopmental disorders in the associations between externalizing behaviors and dementia. Fourth, we were only able to follow most of the individuals until their seventies, whereas the onset of dementia usually peaks around 80 years of age (McMurtray., Clark, Christine, & Mendez, 2006). Fifth, despite ascertaining data from Swedish registers having important advantages, a possible limitation to be considered is a potential misclassification of some dementia cases recorded in the NPR and the CDR (Zhang et al., 2021). Sixth, in the present study we do not control whether externalizing behaviors in the index person occurred before the diagnosis of AD or dementia in the relatives since we aimed to include as many cases as possible to increase the power of our analysis. Nevertheless, the possibility of the onset of dementia in the relatives occurring before the externalizing behaviors in the index person is low since the onset of dementia occurs later on in the life of the relatives and the externalizing behaviors included in this study (criminal behavior and SUD) tend to peak in later adolescence or early adulthood. Therefore, considering the nature of our study design and the studied conditions, it is unlikely that this would significantly affect our associations. Finally, externalizing behaviors (i.e., criminal behaviors, substance abuse) recorded in the NCR and the NPR may represent the most severe cases of the externalizing behavioral spectrum (Kendler et al., 2016). The majority of criminal acts may not end up in a criminal conviction and not all individuals with substance misuse or abuse seek clinical health support where SUD is established. Therefore, their criminal behaviors or diagnosis of SUD may never be recorded in the registers.

## Conclusion

We found a familial coaggregation between externalizing problems with AD and any dementia in prior generations. Our results provide initial evidence of a link between externalizing and criminal behavior and the onset of neurodegenerative disorders in prior generations. Our results may help to develop preventive interventions to reduce the development of dementia among psychiatric and forensic individuals. Further investigation is needed to explore the associations between externalizing problems and dementia and, if verified, more investigation to understand the association and to develop preventive strategies. Further familial studies are warranted to examine common familial risk factors that may contribute to the development of both externalizing problems and dementia.

## Supplementary Material

igad117_suppl_Supplementary_Material

## Data Availability

Restrictions apply to the availability of the data that supports the findings of this study. The Public Access to Information and Secrecy Act in Sweden prohibits individual-level data to be publicly available. Researchers who are interested in replicating this study can apply for individual-level data at Statistics Sweden: https://www.scb.se/en/services/ordering-data-and-statistics/. This study was not preregistered.
