# Supplementary material for: Externalizing Behaviors and Alzheimer’s Disease and Any Dementia: A Multigeneration Cohort Study in Sweden
Source: Innov Aging. 2023 Oct 9;7(9):igad117. doi: 10.1093/geroni/igad117 (PMC10652177; doi:10.1093/geroni/igad117)
Supplement: igad117_suppl_Supplementary_Material [file igad117_suppl_supplementary_material.docx]

**Externalizing behaviors and Alzheimer’s Disease and any dementia: a multi-generation cohort study in Sweden**

Carmen Solares, MSc, Le Zhang, MSc, Zheng Chang, PhD, Henrik Andershed , PhD, Jonas Persson, PhD, & Henrik Larsson, PhD

**Online Supplementary Material**

Table S1. OUTCOMES: Alzheimer´s Diseases and any Dementia codes.

Table S2. Sensitivity analyses of familial coaggregation between externalizing behaviors and any dementia.

Figure S1. Directed Acyclic Graph of the association between Externalizing behaviors and AD and Dementia with extended families.

Table S3. Percentage of externalizing problems and dementia and AD cases among the female and the male populations.

Table S4. ICD codes for Substance Abuse Disorder from the National Patient Register.

**Table S1.** OUTCOMES: Alzheimer´s Diseases and any Dementia codes

| **Outcome** | **ICD-7**†  (1964-1968) | **ICD-8**†  (1969-1986) | **ICD-9**†  (1987-1996) | **ICD-10**†  (1997-2013) | **ATC**‡  (2005-2014) |
| --- | --- | --- | --- | --- | --- |
| **Alzheimer´s disease (AD)** | 304-305 | 290 | 290A/B/X, 331A | F00, F03, G30 | N06DA02-N06DA04, N06DX01 |
| **Vascular dementia** | 306 | 293.0-293.1 | 290E | F01 | - |
| **Other dementia** | - | - | 294B, 290W, 331B/C/X | F02, F02.1, F02.2, F02.3, F02.4, F02.8, F05.1, G31.1, G31.8, F02.8 | - |

NOTE. † ICD Codes for Dementia diagnosis from the NPR and the CDR. ‡ ATC codes from PDR.

**Table S2.** Sensitivity analyses of familial coaggregation between externalizing behaviors and any dementia.

| Analysis | Type of relative | N. pairs | N. events | Non-violent crime | Violent crime | SUD |
| --- | --- | --- | --- | --- | --- | --- |
|  |  |  |  | Adjusted HRs (95%CI) | Adjusted HRs (95%CI) | Adjusted HRs (95%CI) |
| Additional Adjustment for externalizing problems in the relatives of the index person | Parents | 3,364,614 | 15,315 | 1.12 (1.07-1.17) | 1.26 (1.15-1.37) | 1.16 (1.07-1.25) |
|  | Grandparents | 7,951,101 | 808,428 | 1.04 (1.04-1.05) | 1.06 (1.05-1.08) | 1.06 (1.05-1.08) |
|  | Uncles/aunt | 2,443,726 | 11,783 | 1.05 (1.00-1.12) | 1.15 (1.04-1.27) | 0.99 (0.89-1.09) |
| Including medication in addition to diagnosis and cause of death for the case identification | Parents | 3,364,614 | 17,943 | 1.15 (1.10-1.20) | 1.34 (1.23-1.45) | 1.27 (1.18-1.37) |
|  | Grandparents | 7,951,101 | 859,624 | 1.04 (1.04-1.05) | 1.06 (1.05-1.08) | 1.07 (1.05-1.08) |
|  | Uncles/aunt | 2,443,726 | 14,044 | 1.07 (1.02-1.13) | 1.17 (1.06-1.30) | 1.04 (0.95-1.15) |

Note: Abbreviation: HRs, Hazard Ratios; CI, Confidence interval. SUD, substance use disorder. N of events are shown for index person-relative pair (not individuals); Adjusted Hazard Ratios (HRs) were derived from cox proportional models adjusted by birth year of index person, birth year of relatives, sex of index person and sex of relatives.

**Figure S1.** Directed Acyclic Graph of the association between Externalizing behaviors and AD and Dementia with extended families.

C

Externalizing behaviors *Index P*

Externalizing behaviors *Relatives*

AD/Any d*ementia*

*Index P*

AD/Any d*ementia*

*Relatives*

U *Index P*

U *Relatives*

U AD/Any d*ementia*

U E*xternalizing behaviors*

a

a

a

a

b

c

b

b

c

c

c

d

a

Figure S1 illustrates the possible underlying mechanisms of the familial co-aggregation between Externalizing behaviors and AD and any Dementia by a Directed Acyclic Graph (DAG). In the graph C denotes genetic and familial risk factors common for Externalizing behaviors, AD and any Dementia shared by both the index person and his/her relatives. “U externalizing behaviors” represent common causes for externalizing behaviors alone, independent of C. “U AD/any dementia” represent common causes for AD/dementia alone, independent of C. “U Index P” and “U Relatives” denotes individual specific risk factors for externalizing behaviors, AD and any dementia respectively. We hypothesized the following underlying mechanisms (denoted by pathways a, b, c, d) in the current study contributing to the observed familial co-aggregation between externalizing behaviors in the index person and dementia in his/her relatives.

Path a: Externalizing behavior index person ← C → AD/any dementia relatives, where C are genetic and familial risk factors affecting both conditions.

Path b: Externalizing behavior index person ← U Externalizing behaviors → Externalizing behavior relatives → dementia relatives, where genetic and familial risk factors for Externalizing behaviors mediate the risk of having dementia. Though this mechanism genetic and familial risk factors increase the risk of having Externalizing behaviors in both index person and his/her relatives, and the risk of having dementia in relatives is increased by the adverse health and psychosocial outcomes triggered by their externalizing behaviors.

Path c: Externalizing behavior index person ← Externalizing behavior relatives ← U relatives → dementia relatives or externalizing behavior index person ← Externalizing behavior relatives → dementia relatives. In this pathway, the association between externalizing behaviors in the index person and dementia in relatives is explain by carry-over effects (Externalizing behavior index person ← Crime/externalizing behavior relatives). This mechanism suggests a direct effect of externalizing behaviors in the relatives on externalizing behaviors in the index person, as well as on the risk of dementia. For example, poor parental skills or more unstable familiar relationships in individuals with externalizing behaviors may influence the behavior of their children who may explain the coaggregation in the index-parent cohort (but the coaggregation with more distant relative cohorts may not be explain with this pathway).

Path d: Externalizing behavior index person → dementia relatives: This pathway denotes a direct effect of externalizing behaviors in the index person on the development of dementia in the relatives. Although there is no evidence suggesting a direct evidence for this, a possibility is that parents of children who engage in crime/externalizing behaviors may experience depression and psychological distress which my increase their risk of developing dementia. Although this could potentially explain associations in the cohort of index person and parents, this mechanism explains less likely the associations with more distant relatives.

**Table S3**. Percentage of externalizing problems and dementia and AD cases among the female and the male populations.

| Type of individual | Variable | Female | Male |
| --- | --- | --- | --- |
| **Index Person** | **N of index person** | **1,198,443** | **1,264,590** |
|  | Non-violent crime, N. (%) | 86,102 (7.18) | 207,776 (16.43) |
|  | Violent crime, N. (%) | 13,070 (1.09) | 73,835(5.83) |
|  | SUD, N. (%) | 48,443 (4.04) | 64,083 (5.06) |
| **Parents** | **N of parents** | **1,303,234** | **1,298,127** |
|  | Alzheimer Disease, n. (%) | 3,348(0.25) | 5,959(0.45) |
|  | Any dementia, n. (%) | 4,024 (0.30) | 7,787 (0.59) |
| **Grandparents** | **N of grandparents** | **1,284,719** | **1,344,629** |
|  | Alzheimer Disease, n. (%) | 133,574 (10.39) | 87,482 (6.5) |
|  | Any dementia, n. (%) | 149,676 (11.65) | 103,926 (7.72) |
| **Uncles/Aunts** | **N of uncles/aunts** | **784,895** | **755,934** |
|  | Alzheimer Disease, n. (%) | 2,965 (0.37) | 2,634 (0.34) |
|  | Any dementia, n. (%) | 3,487 (0.44) | 3,493 (0.46) |

**Table S4**. ICD codes for Substance Abuse Disorder from the National Patient Register.

| **Covariate** | **ICD-8**†  (1969-1986) | **ICD-9**†  (1987-1996) | **ICD-10**†  (1997-2013) |
| --- | --- | --- | --- |
| **Substance use disorder** | 303, 304 | 303, 304, 305 | F10 – F19 |
